# The Contribution of Psychological Wellbeing and Emotion-Regulation to Foreign Language Teaching Enjoyment

**DOI:** 10.3389/fpsyg.2022.889133

**Published:** 2022-05-02

**Authors:** Mostafa Azari Noughabi, Neda Fekri, Fatemeh Kazemkhah Hasankiadeh

**Affiliations:** ^1^Department of English Language and Literature, Gonabad University, Gonabad, Iran; ^2^Department of English Language, Aliabad Katoul Branch, Islamic Azad University, Aliabad Katoul, Iran; ^3^Department of English Language, Ferdowsi University of Mashhad, Mashhad, Iran

**Keywords:** foreign language teaching enjoyment (FLTE), psychological wellbeing, emotion regulation, EFL teachers, structural equation modeling (SEM)

## Abstract

The notion of foreign language teaching enjoyment (FLTE) has recently been introduced to the field of foreign language education as a positive emotion which influences teachers’ professional and psychological growth. Due to the pure novelty of the concept, scant research attention has been paid to its antecedents in English as a foreign language (EFL) contexts. The present study aims to investigate the extent to which psychological wellbeing and emotion regulation can contribute to FLTE of Iranian EFL teachers. The participants (*N* = 575) responded to three electronic questionnaires and the collected data were then analyzed through structural equation modeling (SEM). The SEM analysis indicated that Iranian EFL teachers’ psychological wellbeing and emotion regulation significantly influenced their FLTE. The findings revealed that psychological wellbeing was a stronger predictor of EFL teachers’ FLTE in the Iranian context. The findings were discussed regarding the causes of increasing teaching enjoyment. The results highlighted the importance of applying psychology in language teaching research. Finally, implications and suggestions for future research are offered.

## Introduction

Language teaching is an emotional profession (Mercer, 2020). Accordingly, language teachers’ emotions should be taken into account to increase psychological and professional flourishing (Benesch, 2018). With the burgeoning of positive psychology in the field of foreign language education (Dewaele et al., 2019), researchers have shifted their attention from deciphering negative factors such as teacher stress and burnout to assessing different positive variables such as wellbeing, engagement, and positive emotions (Mercer, 2020; Fathi and Mohammaddokht, 2021; Greenier et al., 2021). Research has shown that positive emotions impact teachers’ professional practices and learners’ engagement (Hagenauer et al., 2015). Moreover, the key role of positive emotions in language teaching has been already well documented (Mercer, 2020). Language teachers’ psychological growth and professional success can be enhanced by their experience of positive emotions such as enjoyment (Gregersen and MacIntyre, 2021).

The notion of foreign language teaching enjoyment (FLTE), introduced by Proietti Ergün and Dewaele (2021), refers to a positive emotion which plays a pivotal role in language teachers’ profession. According to Proietti Ergün and Dewaele (2021), foreign language teachers with high FLTE feel a high rate of job satisfaction and happiness. In other words, mentally happy language teachers are less likely to experience burnout, emotional exhaustion, and attrition. In their concluding remarks, Proietti Ergün and Dewaele (2021) noted the need for conducting further studies on FLTE and its correlates. Therefore, it is of importance to identify which psychological variables can contribute to EFL teachers’ enjoyment.

Two possible related positive factors that can affect FLTE are emotion regulation and psychological wellbeing. Emotion regulation refers to one’s adoption of strategies to change, control, or manage their emotions in order to ensure the accomplishment of their goals (Sutton, 2004). Research shows that language teachers who can regulate their emotions enjoy higher levels of job efficiency, work engagement, and wellbeing (Bielak and Mystkowska-Wiertelak, 2020; Greenier et al., 2021). In addition to emotion regulation, wellbeing has been regarded as an important factor influencing teachers’ pedagogical practices and psychological growth (Mercer and Gregersen, 2020). Research shows that language teachers with high levels of wellbeing are more effective and delighted (Proietti Ergün and Dewaele, 2021).

Many previous studies have focused on students’ foreign language enjoyment (Dewaele and MacIntyre, 2019; Li, 2020; Fathi and Mohammaddokht, 2021; Jin and Zhang, 2021), highlighting the relationship between EFL learners’ foreign language enjoyment (FLE) with their foreign language performance (Jin and Zhang, 2019; Wei et al., 2019), subjective wellbeing (Proietti Ergün and Ersöz Demirdağ, 2022), and engagement (Guo, 2021). Despite increasing number of studies on language learners’ FLE, language teacher enjoyment is rarely researched. In spite of the significant role of enjoyment in teachers’ psychological growth, research on FLTE is at nascent stages (Proietti Ergün and Dewaele, 2021). In effect, examining teachers’ FLTE and its correlates has remained less explored (Proietti Ergün and Dewaele, 2021). Particularly, identifying its psychological sources and their casual relationships with FLTE through structural equation modeling (SEM) has remained under shadow. Therefore, the present study aims to investigate whether psychological wellbeing and emotion regulation can predict FLTE among EFL teachers in the context of Iran. The results of the study can shed more light on positive psychology of EFL teachers through indicating the factors which enhances their enjoyment. This study addresses the following research question.

To what extent do psychological wellbeing and emotion regulation contribute to teachers’ FLTE in the EFL context of Iran?

## Literature Review

With the advent of positive psychology in foreign language education (Budzińska and Majchrzak, 2021; Greenier et al., 2021), researchers began to study positive factors (Seligman and Csikszentmihalyi, 2014; Wang et al., 2021) among which FLTE is the main focus of the present study. Structured on the “broadening-and-building” nature of positive emotions highlighted by Fredrickson (2001), positive feelings broaden the teachers’ awareness and motivate creative thoughts and actions. Regarding the novelty of the concept of FLTE as a critical aspect of EFL teachers’ personal and professional lives (Proietti Ergün and Dewaele, 2021), studies on the contributors to FLTE deserve more attention.

### Foreign Language Teaching Enjoyment

FLTE has been termed by Proietti Ergün and Dewaele (2021) as a positive emotion which impacts EFL teachers’ psychological state and their learners’ linguistic growth. FLTE is composed of three domains, naming social enjoyment, personal enjoyment, and student appreciation. Given that enjoyment is “a complex emotion, capturing interacting dimensions of challenge and perceived ability to reflect human drive for success in the face of difficult tasks” (Dewaele and MacIntyre, 2016, p. 216), identifying the psychological factors which can contribute to higher levels of enjoyment and success is of utmost importance. In the present study, FLTE refers to EFL teachers’ positive emotion composed of their personal and social enjoyment plus their feeling of being appreciated by the students.

Despite numerous studies on students’ foreign language enjoyment (Dewaele and MacIntyre, 2016, 2019; Dewaele et al., 2018; Li et al., 2018; Jin and Zhang, 2019; Dewaele and Dewaele, 2020; Li, 2020; Botes et al., 2021), teachers’ FLTE has scarcely been probed suggesting that studying FLTE and its correlates is at early stages. In one of the rare studies on teaching enjoyment, Mierzwa (2019) reported a relatively high level of teaching enjoyment among 89 Polish teachers of different languages regardless their gender, place of residence, years of experience, school type, and the type of foreign language they taught. In her concluding remarks, Mierzwa highlighted the key role of teachers’ positive emotions in foreign language classrooms which influences “the learners’ attitude to life-long learning” (p. 185). In spite of the contribution of her study, still the findings cannot be generalized due to the small sample size. Examining the underlying dimensions of foreign language classroom enjoyment in a Chinese secondary school context, Jin and Zhang (2021) found that enjoyment of student support coupled with enjoyment of teacher support positively influenced foreign language learners’ enjoyment of English learning which subsequently predicted their language achievement. In another study, Jiang and Dewaele (2019) have highlighted the strong effect of teacher-related variables on language learners’ foreign language enjoyment. The results of their mixed method study on 564 Chinese undergraduate EFL learners implied that the learners’ awareness of enjoyment was mostly triggered by their teachers.

In a pioneering study on 174 Italian foreign language teachers, Proietti Ergün and Dewaele (2021) designed a 9-item scale for measuring FLTE and investigated the relationship between mental wellbeing, resilience, and FLTE. They identified mental wellbeing and resilience as two significant correlates of FLTE. The findings of their study indicated that happy and resilient teachers can create a positive emotional atmosphere in the classroom which might be regarded as a prerequisite for learners’ linguistic and teachers’ psychological development. In their conclusion, Proietti Ergün and Dewaele (2021) stressed the need to conduct further studies on correlates of FLTE. To pinpoint what shapes the novel psychological concept of FLTE, the present study aims to find out the extent to which EFL teachers’ emotion regulation and psychological wellbeing impact FLTE. Though the present study is inspired by Proietti Ergün and Dewaele’s (2021) analysis, still appointing a SEM approach and including a far larger sample size guarantees the expected contribution of their study.

### Emotion Regulation

Gross (1998) conceptualized emotion regulation as an amalgamation of different processes for controlling or expressing feelings. According to Gross and John (2003), there are two broad categories of the strategies one can apply to regulate his or her emotions: cognitive reappraisal and expressive suppression strategies. While expressive suppression refers to the inhabitation of external cues to one’s internal emotional state, cognitive reappraisal involves “construing a potentially emotion eliciting situation in a way that changes its emotional impact” (Gross and John, 2003, p. 349). In the present study, emotion regulation is operationalized as EFL teachers’ employment of cognitive reappraisal and expressive suppression strategies to manage their emotions in the classroom context.

Lee et al. (2016) maintains that “teachers experience a variety of emotions on a daily basis and how they manage these emotions is crucial not only for their own emotional wellbeing, but also for their professional performance in educational settings” (p. 844–845). Scholars have highlighted emotion regulation as a required task for effective teaching (Ghanizadeh and Royaei, 2015; Fathi et al., 2021). Previous studies have indicated the advantages of regulating emotions. For example, Sutton and Harper (2009) point that a teacher who employs emotion regulation strategies can be better at having interpersonal relations with the students. Moreover, as pointed out by Talbot and Mercer (2018), language teachers who can control emotional stressors and hardships in their job are more likely to experience wellbeing.

To date, a series of studies have explored the application of emotion regulation in different contexts. Morris and King (2018) studied emotion regulation strategies of seven EFL university teachers in Japan and found that university teachers regulate their emotions contextually to accomplish their perceived goals. Their applied emotional strategies were effective while dealing with low-level stressors such as students’ apathy and silence and less effective in the case of deliberate stressors such as students’ misbehavior. Another line of inquiry has been focused on correlates of language teachers’ emotional regulation. Studying 153 Iranian EFL teachers working in private sector, Ghanizadeh and Royaei (2015) found a linkage between teachers’ emotion regulation, emotion labor strategies, and teacher burnout. They reported how emotion regulation and emotion labor strategies can lead to teacher burnout, though negatively. In their SEM study on 256 EFL teachers, Fathi and Derakhshan (2019) found that self-efficacy and emotion regulation affected Iranian EFL teachers’ stress.

In sum, existing studies have documented the beneficial role of language teachers’ utilization of emotional regulation strategies in developing their psychological wellbeing. The centrality of emotions in language teaching also signifies the importance of mastering emotion regulation skills (Benesch, 2017). However, studies on EFL teachers’ emotion regulation are at early stages (Greenier et al., 2021). Therefore, fresh studies are needed to explore the possible psychological correlates and outcomes of emotion regulation.

### Psychological Wellbeing

Dagenais-Desmarais and Savoie (2012) believe that there is no consensus regarding a unified definition for the concept of psychological wellbeing. According to Thorsteinsen and Vittersø (2020), the term psychological wellbeing should be discussed in the light of three approaches: hedonic (i.e., psychological wellbeing is linked with happiness), eudaimonic (psychological wellbeing is associated with optimal functioning), or a combined interactive approach. Ryff (1989), conceptualizes psychological wellbeing as a combination of six elements: self-acceptance; purpose in life, environmental mastery, positive relations with others, autonomy, and personal growth. Psychological wellbeing can be described as one’s appreciation of his or her mental health, pleasure, and satisfaction (Huppert, 2009). Accordingly, teachers’ psychological wellbeing can be conceptualized as “teachers’ satisfaction with their daily working environment” (Sisask et al., 2014, p. 384). In the current study, psychological wellbeing refers to EFL teachers’ perceived competence, success at work, involvement to the job, and interpersonal fit at work.

One major line of inquiry has been dedicated to the exploration of the factors which influence or are influenced by language teachers’ wellbeing. According to Talbot and Mercer (2018), low self-confidence and limited emotion regulation would negatively impact language teachers’ wellbeing. In a recent study, Proietti Ergün and Dewaele (2021) found a positive correlation between foreign language teachers mental wellbeing and teaching enjoyment. In their cross-cultural study, Greenier et al. (2021) figured out that psychological wellbeing was a significant predictor of both Iranian (*N* = 255) and British (*N* = 108) language teachers’ work engagement. Likewise, Kong (2021) studied 304 Chinese EFL teachers’ work engagement and found that their self-efficacy and psychological wellbeing significantly predicted their dedication to the job.

Wellbeing has been shown to exert a significant influence on language teachers’ instructional quality and learners’ educational achievement (Talbot and Mercer, 2018; Oxford, 2020). Regarding the complexities and adversities embedded in foreign language teaching, investigating language teachers’ perceived psychological wellbeing deserves attention to rectify the emotional stressors and psychological demands (King and Ng, 2018; MacIntyre et al., 2019). Language teachers with high levels of wellbeing have a positive rapport with learners which can increase their teaching success, happiness, and achievement (Mercer, 2020). Moreover, psychologically happy language teachers who can regulate their emotions experience higher engagement and enjoyment (Greenier et al., 2021). Therefore, it is hypothesized that language teachers’ wellbeing and emotion regulation can contribute to their FLTE.

In sum, reviewing the related literature suggests that there is a scarcity of studies on psychological causes of teachers’ FLTE in EFL contexts. In particular, to the best knowledge of the researchers, no empirical study to date has tested a structural model to identify the causal relationships among EFL teachers’ psychological wellbeing, emotion regulation, and FLTE. Therefore, inspired by positive psychology in foreign language education, the present study seeks to employ SEM to unveil the interplay among psychological wellbeing, emotion regulation, and FLTE among Iranian EFL teachers to expand the line of research focusing on positive teacher-related variables. The aim of the present study is to target the nascent line of inquiry on the predictors of FLTE (Proietti Ergün and Dewaele, 2021) to encourage further related investigations in the arena of language education in various contexts.

## Materials and Methods

### Participants

In this study, a sample of 575 Iranian EFL teachers participated in completing three electronic questionnaires on psychological wellbeing, emotion regulation, and FLTE. They were from diverse geographical areas of Iran. The majority of the participants (73.9%) have been educated in English Language Teaching. The participants were from three work contexts (i.e., university, school, and institute). The teachers were from a wide range of teaching experience from 1 to 30 years of teaching (*M* = 6.84, *SD* = 5.07). Their age ranged from 19 to 61 (*M* = 28.47, *SD* = 6.26). Informed consent was given from all the participants. The demographic information of the participants is presented in Table 1.

**TABLE 1:** Demographic information of the participants.

Gender	Major	Academic degree
Male = 238	English language literature = 94	Bachelor of Arts = 326
Female = 337	English language translation = 148	Master of Arts = 222
	English language teaching = 331	Ph.D. = 18
*N* = 575	Linguistics = 2	Other = 9

### Instruments

#### Foreign Language Teaching Enjoyment Scale

In the present study, the Foreign Language Teaching Enjoyment Scale (FLTES), designed by Proietti Ergün and Dewaele (2021), was used as a reliable measure of teachers’ FLTE. The scale has 9 items on a 5-point Likert scale ranging from 1 (strongly disagree) to 5 (strongly agree). The FLTES includes three subfactors including personal enjoyment (e.g., “I enjoy it.”), student appreciation (e.g., “The students are friendly.”), and social enjoyment (e.g., “We laugh a lot.”). In the present study, the internal consistency of the FLTES calculated via Cronbach’s alpha was 0.90.

#### Emotion Regulation Scale

To measure emotion regulation of the respondents, the Emotion Regulation Scale was used (Gross and John, 2003). The scale consists of 10 items on a 7-point Likert scale ranging from 1 (strongly disagree) to 7 (strongly agree). This scale has two subfactors: Cognitive Reappraisal (e.g., “I control my emotions by changing the way I think about the situation I’m in.”) and Expressive Suppression (e.g., “I control my emotions by not expressing them.”). Previous studies have indicated that the Emotion Regulation Scale is a reliable and valid for measuring EFL teachers’ emotion regulation (Greenier et al., 2021). In the present study, this scale enjoyed good internal consistency (α = 0.90).

#### Psychological Wellbeing Scale

To assess the participants’ psychological wellbeing, the Psychological Wellbeing at Work (PWBW), designed by Dagenais-Desmarais and Savoie (2012), was used. This scale contains 25 items on a 6-point Likert scale ranging from 0 (disagree) to 5 (completely agree). The PWBW includes five subfactors including interpersonal fit at work (e.g., “I enjoy working with the people at my job.”), thriving at work (e.g., “I am proud of the job I have.”), feeling of competency at work (e.g., “I feel confident at work.”), perceived recognition at work (e.g., “I feel that the people I work with recognize my abilities.”), and desire for involvement at work (e.g., “I care about the good functioning of my organization.”). Research indicates that the PWBW is a valid measure of psychological wellbeing (Sandilya and Shahnawaz, 2018). In the current study, the reliability of the PWBW estimated via Cronbach’s alpha was 0.97.

### Data Collection

Data were collected in January 2022 from EFL teachers in Iran through three online electronic forms designed via Google Forms. Convenience and snowball sampling were employed to recruit the participants. Survey link was shared with the participants via WhatsApp and Telegram groups. Before completing the questionnaire, informed consent was given from the respondents. Instructions for completing the forms were attached and no deadline was set to send back the completed forms. The participants were reminded to mark their responses regardless of their experiences during the COVID-19 pandemic. The participants were assured that their confidentiality will be considered.

### Data Analysis

Iinitially, the data were screened out, using SPSS 26, to check the possibility of missing data, outliers, and multicollinearity. After ensuring these issues, confirmatory factor analysis (CFA) and SEM analysis were carried out via AMOS 26. As for model fit indices, the chi-square (χ^2^), comparative fit index (CFI), goodness of fit (GFI), adjusted goodness of fit index (AGFI), Tucker-Lewis index (TLI), root mean square error of approximation (RMSEA), and standardized root mean square residual (SRMR) were considered (Hu and Bentler, 1995).

## Results

This section reports the results of CFA and SEM analysis to answer the research question regarding the psychological antecedents of Iranian EFL teachers’ FLTE. Prior to CFA and SEM analyses, descriptive statistics with correlation between the main variables were calculated. Table 2 shows descriptive statistics, reliability of the scales, and zero correlations between the variables. As shown in Table 2, the main variables are significantly correlated.

**TABLE 2:** Descriptive statistics, reliability, and correlations between analyzed variables.

Construct	*M*	*SD*	α	Skewness	Kurtosis	1	2	3
**EMR**	2.99	0.81	0.90	2.09	4.27	1		
**PWB**	2.88	0.73	0.97	0.52	0.76	0.59**	1	
**FLTE**	3.05	0.60	0.90	1.10	1.50	0.66**	0.87**	1

PWB, Psychological Wellbeing; FLTE, Foreign Language Teaching Enjoyment; EMR, Emotional Regulation, **p < 0.01.

Next, CFA was administered to ensure the construct validity of all the scales. The aforementioned goodness-of-fit indices were taken into account to check the measurement models. As shown in Table 3, the models verified a good fit to the data. Therefore, the CFA results verified the factor structure of the observed variables suggesting that a significant relationship between observed variables and their underlying latent constructs exists.

**TABLE 3:** Goodness of fit measures for the research constructs.

Constructs	χ^2^/df	GFI	AGFI	TLI	CFI	SRMR	RMSEA
**EMR**	3.02	0.97	0.94	0.98	0.98	0.03	0.06
**PWB**	3.01	0.90	0.88	0.94	0.95	0.03	0.06
**FLTE**	2.02	0.98	0.96	0.98	0.99	0.02	0.05

*PWB, Psychological Wellbeing; FLTE, Foreign Language Teaching Enjoyment; EMR, Emotional Regulation.*

**FIGURE 1:**
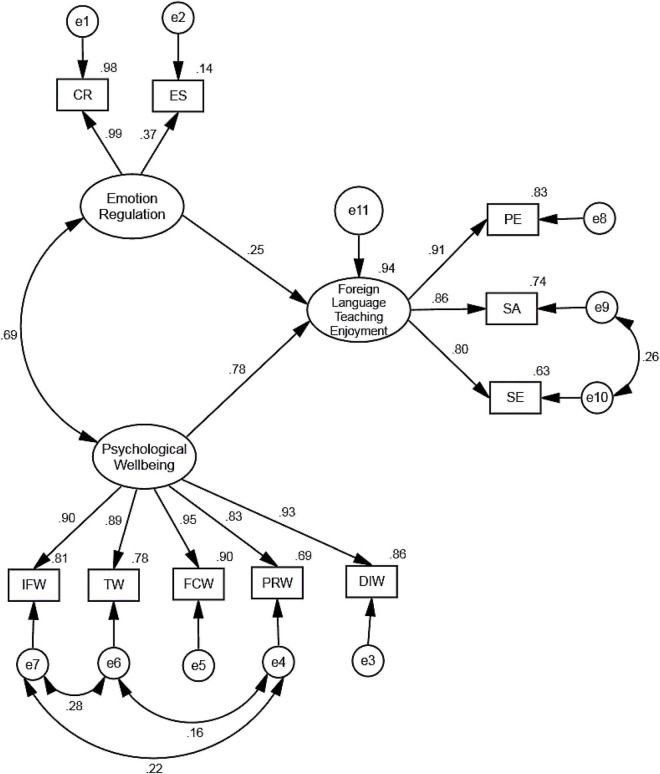
The SEM model of the association between EFL teachers’ FLTE, psychological wellbeing, and emotion regulation. CR, Cognitive Reappraisal; ES, Expressive Suppression; IFW, Interpersonal Fit at Work; TW, Thriving at Work; FCW, Feeling of Competency at Work; PRW, Perceived Recognition at Work; DIW, Desire for Involvement at Work; PE, Personal Enjoyment of FL Teaching; SA, Student Appreciation in the FL Classroom; SE, Social Enjoyment of FL Teaching.

Following the CFA, the hypothesized SEM model was tested with maximum likelihood procedure. To this end, one unidirectional path from emotion regulation to FLTE, one unidirectional path from psychological wellbeing to FLTE, and a bidirectional path between emotion regulation and psychological wellbeing were drawn and error terms were also added to SEM model. Initially, the achieved fit indices were in an acceptable range except RMSEA which was found to be 0.98. Next, based on suggested modifications, we correlated four error terms. As shown in Figure 1, the modified model indicated good model fit (df = 29, χ^2^ = 94.680, χ^2^/df = 3.265, CFI = 0.989, TLI = 0.982, RMSEA = 0.063, SRMR = 0.026, *p* < 0.001) and the coefficient for all paths were significant (*p* < 0.001). Therefore, the results of SEM confirmed fitness of the hypothesized model (Kline, 2011).

As demonstrated in Figure 1, Iranian EFL teachers’ FLTE was significantly influenced by their psychological wellbeing (β = 0.78, *p* < 0.001) and emotion regulation (β = 0.25, *p* < 0.001). Also, SEM results indicated that psychological wellbeing had the greatest effect on Iranian EFL teachers’ FLTE. Furthermore, emotion regulation and psychological wellbeing were found to be positively correlated (β = 0.69, *p* < 0.001).

## Discussion

The current study aimed to assess the contribution of EFL teachers’ psychological wellbeing and emotion regulation to their FLTE in the context of Iran. The results of SEM analysis indicated that Iranian EFL teachers’ psychological wellbeing and emotion regulation significantly influenced their FLTE. Psychological wellbeing was also found as a stronger predictor of FLTE among Iranian EFL teachers. The findings of the present study can be justified based on the tenets of positive psychology in foreign language education (Dewaele et al., 2019; Wang et al., 2021) suggesting that improving EFL teachers’ psychological strengths and wellbeing can result in positive outcomes. From a theoretical viewpoint, the findings showcase the significance of applying positive psychology in foreign language teaching, suggesting that EFL teachers’ positive experiences (i.e., emotion regulation and psychological wellbeing) can enhance their FLTE which is a desirable positive emotion for language teachers and language learners alike (Proietti Ergün and Dewaele, 2021).

The significant influence of emotion regulation on FLTE can be justified referring to Hiver (2017) who notes that regulating emotions is necessary to experience satisfaction and stay immune to professional hurdles. This finding suggests that emotional regulatory skills are needed for EFL teachers, who are surrounded with a bewildering range of professional challenges and psychological stressors (Borg, 2006; Mercer, 2020), to enjoy the profession and teach with love (Proietti Ergün and Dewaele, 2021). This finding highlights the importance of emotions in EFL teachers’ professional engagement and psychological development (Noughabi et al., 2020). This finding supports the study of Greenier et al. (2021) which depicts that EFL teachers with high levels of psychological wellbeing and emotional regulation are more engaged in their job and experience higher levels of enjoyment and job satisfaction (Burić and Macuka, 2018).

The significant association between EFL teachers’ psychological wellbeing and FLTE is in accord with the findings reported by Proietti Ergün and Dewaele (2021). In effect, the present findings complement the correlational findings of the study of Proietti Ergün and Dewaele (2021) by indicating the causal influence of EFL teacher’ wellbeing on their teaching enjoyment. The greater impact of psychological wellbeing on FLTE can be attributed to the fact that psychologically happy and resilient language teachers can sustain their positive feelings despite daily stressors (Wang et al., 2022). Therefore, resilient, immune teacher can regulate their emotions especially in case of adversities; consequently, they experience higher levels of wellbeing (Hiver, 2018; Noughabi et al., 2020). Thus, happy teachers’ utilization of successful emotion regulation strategies can impact their wellbeing, dedication, and self-efficacy (Azari Noughabi and Amirian, 2021; Greenier et al., 2021) that leads them to teaching enjoyment.

The positive correlation between EFL teachers’ emotion regulation and psychological wellbeing further supports previous studies (e.g., Greenier et al., 2021; Li, 2021) suggesting that EFL teachers who are more competent in regulating their emotions in the classroom context are more likely to experience happiness during their careers. This finding corroborates the idea of Ghanizadeh and Royaei (2015) who believe that EFL teachers who can manage their emotions are less likely to experience burnout and frustration. Therefore, in line with previous studies (e.g., Talbot and Mercer, 2018; Bielak and Mystkowska-Wiertelak, 2020), the findings revealed that EFL teachers’ psychological wellbeing might hinge upon their ability to sustain their positive feelings and control their negative emotions (Morris and King, 2018).

In sum, the findings of this study are in line with recent studies indicating the key role of emotion regulation and psychological wellbeing in language teachers’ psychological health and emotional growth (Gregersen et al., 2020; Mercer and Gregersen, 2020). Happy EFL teachers who employ proper regulatory skills to control their emotions are more likely to accomplish their professional objectives which leads them to more energy, love, and enjoyment (Greenier et al., 2021). Subsequently, EFL teachers enjoy creating a positive atmosphere in the classroom which facilitates both their own healthy psychological functioning and their learners’ linguistic performance (Proietti Ergün and Dewaele, 2021; Yang et al., 2022). In response to the need to conduct further studies on the novel concept of FLTE and its correlates, this study provides insight into the positive antecedents of enjoyment in foreign language teaching as framed by the tenets of positive psychology in foreign language education. Unlike previous studies on students’ foreign language enjoyment, the current study focused on foreign language teachers’ enjoyment which can contribute to the field of language teacher education by identifying the predictors of teachers’ FLTE. In addition, a rare SEM study can contribute to the ongoing discussions in literature regarding the influence of positive psychological variables on the manifestation of teaching enjoyment.

## Conclusion

This study targeted to contribute to the dearth of knowledge on the possible predictors of FLTE by identifying the associations between FLTE, emotion regulation, and psychological wellbeing among EFL teachers in the Iranian context. The results of SEM indicated that (a) emotion regulation and psychological wellbeing were significantly correlated (b) emotion regulation and psychological wellbeing were significant contributors to FLTE in the Iranian context, and (c) psychological wellbeing was a stronger predictor of FLTE. The findings of the present study signify that teacher educators can develop language teacher’ FLTE by introducing them ways of sustaining positive emotions and controlling negative ones in order to achieve a state of balance affected by both challenging and rewarding teaching events. Once FLTE is confirmed, the quality of instruction is expected to be boosted as well.

The findings imply that improving teachers’ mastery of skills in response to emotional experiences might be an influential factor in supporting EFL teachers against burdens in the profession. As EFL teachers’ psychological wellbeing was found to be the most significant predictor of FLTE, it is highly suggested to support EFL teachers, whether economically or psychologically, and increase their happiness with the ultimate goal of helping them become immersed in their occupation in a joyful fashion. This goal can be reached by increasing their salary, reducing the burden of heavy schedule, and offering supportive mentoring. Another implication that one way to enhance the quality of teachers’ instructional efficiency is to help them enjoy performing their occupational roles. This objective can be accomplished by creating a positive environment in the classroom through regulating emotions despite job-embedded hurdles.

In sum, the findings of this study are encouraging in investigating how selected behaviors such as rethinking a challenging situation to reduce anger or anxiety, hiding sings of sadness or fear, or focusing on reasons to feel happy or calm from the EFL teachers are so influential in guaranteeing FLTE. Bearing in mind that teachers’ psychological wellbeing is dynamic throughout the profession, we conclude that there is a need to give a constant awareness to EFL teachers regarding the vital concept of FLTE along with the newly found predictors; emotion regulation and psychological wellbeing. To have joyful language teachers, finding effective means to improve their regulatory skills and enhance their mental happiness is critical.

## Limitations and Suggestions for Future Research

The findings of the current study need to be interpreted in light of the following limitations. First, the study presented herein utilized self-report data which possibly may not reflect EFL teachers’ actual perception of their emotion regulation, psychological wellbeing, and FLTE. Methodological triangulation is needed to be done in future studies to provide a better picture of the interaction between these three variables. Second, the present study focused on a sample of EFL teachers who were from Iran. Further multinational studies are needed to test the generalizability of the findings. In addition, cross-cultural studies are needed to evaluate the possible invariances across various cultures. Third, this study did not add demographic variables such as years of teaching experience to the proposed model. Avid researchers are recommended to investigate whether demographic variables can mediate the relationships among the studied variables.

## Data Availability Statement

The raw data supporting the conclusions of this article will be made available by the authors, without undue reservation.

## Ethics Statement

Ethical review and approval was not required for the study on human participants in accordance with the local legislation and institutional requirements. The patients/participants provided their written informed consent to participate in this study.

## Author Contributions

MA directed the study and devised the conceptual framework, data interpretation, manuscript writing, designed the models, and revised the manuscript. NF assisted with the development of conceptual framework and data collection and contributed part of the manuscript writing. FK assisted with data collection and contributed part of the manuscript writing. All authors contributed to the article and approved the submitted version.

## Conflict of Interest

The authors declare that the research was conducted in the absence of any commercial or financial relationships that could be construed as a potential conflict of interest.

## Publisher’s Note

All claims expressed in this article are solely those of the authors and do not necessarily represent those of their affiliated organizations, or those of the publisher, the editors and the reviewers. Any product that may be evaluated in this article, or claim that may be made by its manufacturer, is not guaranteed or endorsed by the publisher.
